# Establishment and characterisation of a human carcinoma cell line with acquired resistance to Aplidin™

**DOI:** 10.1038/sj.bjc.6602166

**Published:** 2004-09-14

**Authors:** A Losada, J M López-Oliva, J M Sánchez-Puelles, L F García-Fernández

**Affiliations:** 1 Drug Discovery Department, PharmaMar, S.A., E-28770-Colmenar Viejo, Madrid, Spain

**Keywords:** natural product, drug resistance, MAPK, apoptosis

## Abstract

Aplidin™ (APL) is a new antitumoral drug from marine origin currently in phase II clinical trials against a wide multiplicity of cancers. As resistance may be, as with other drugs, an important obstacle to the APL therapeutic efficacy, we have established an acquired resistance cellular model by continuous exposure of HeLa cells to the drug. The stably resistant subline generated (HeLa-APL), possessing more than 1000-fold relative resistance to APL than parental cells, did not show crossresistance to a subset of clinically relevant antitumoral agents. In addition, resistance was not related to overexpression of P-glycoprotein or differences in overall drug accumulation. Comparing to parental cells, HeLa-APL cells did not present either significant differences in the growth rate or apparent alterations in the cell cycle distribution. Aplidin™ induced rapid and persistent phosphorylation of both JNK and p38 MAPKs, resulting in activation of the mitochondrial apoptotic pathway in parental cells, but, notably, in HeLa-APL-resistant cells MAPKs activation only occurred in a slight and transiently manner, failing to activate the above-mentioned apoptotic machinery. These results suggest that sustained activation of JNK and p38 is essential for triggering the apoptotic programme induced by APL and that HeLa-APL cells bypass this apoptotic response by preventing the specific mechanisms that prime and sustain the long-term activation of these signalling cascades. Although far from human tumour physiology *in vivo*, HeLa-APL cells represent a potentially useful tool in gaining insights into the mode of action of APL, in selecting non-crossresistant APL structural analogues, as well as in investigating and developing methods to prevent resistance to this drug.

Aplidin™ (APL, a macrocyclic depsipeptide, C_57_H_87_N_7_O_15_) (Figure 1

**Figure 1:**
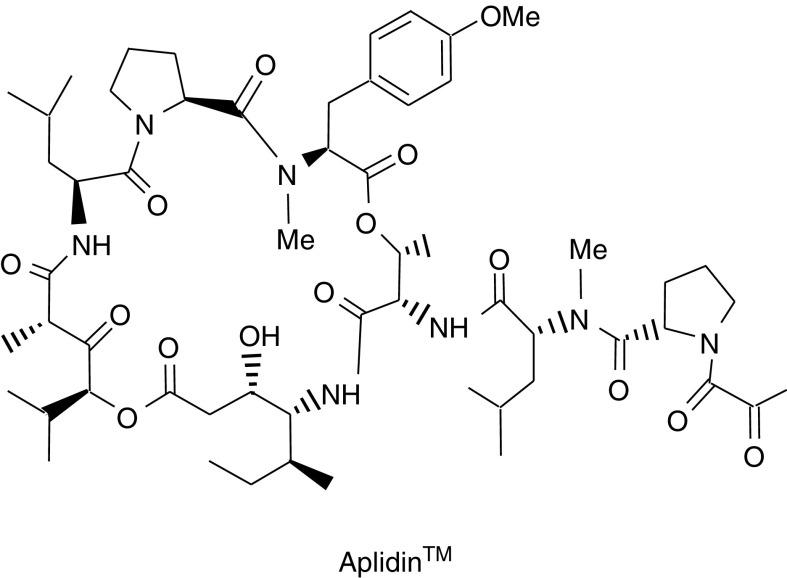
Molecular structure of Aplidin™ (APL).

) is a novel antitumoral agent isolated from the marine tunicate *Aplidium albicans* (Sakai *et al*, 1996; Rinehart, 2000). Aplidin™ was developed as an antineoplastic agent because of its potent antitumour activity in preclinical models against a wide variety of human tumours (Faircloth *et al*, 1998, 1999). Clinical trials with APL were initiated in 1999 in different locations of Europe and Canada. Results from phase I clinical trials showed signs of clinical benefit, in terms of stabilisation of the disease, and a favourable tolerability profile, with mild and relatively infrequent side effects (Anthoney *et al*, 2000; Armand *et al*, 2001; Bowman *et al*, 2001; Mauroun *et al*, 2001; Ciruelos *et al*, 2002). In addition, based on recent results showing that APL presents selective cytotoxicity *in vitro* against childhood leukaemia cells, a phase I clinical trial to study the effect of APL in pediatric leukaemia is under implementation (Bresters *et al*, 2003; Erba *et al*, 2003). Aplidin™ is currently in phase II clinical trials for renal, head and neck, pancreas, lung (NSCLC) and colorectal cancers, and medullary thyroid carcinoma. Recently, both the European Agency for the Evaluation of Medicinal Products (EMEA) and the American Food and Drug Administration (FDA) awarded APL ‘*orphan drug*’ status for the treatment of acute lymphoblastic leukaemia (ALL).

There is little information about the mode of action of the drug in tumour cells. Aplidin™ induces an early oxidative stress response, which results in a rapid and sustained activation of the epidermal growth factor receptor (EGFR), the nonreceptor protein tyrosine kinase *Src*, and the serine/threonine kinases c-jun N-terminal kinase (JNK) and p38 MAPK. These early events rapidly trigger the induction of the mitochondrial apoptotic pathway via cytochrome *c* release, activation of the caspase cascade and activation of protein kinase C (PKC)-*δ*, which seems to exert an important effector role in mediating the cellular death induced by the drug (Garcia-Fernandez *et al*, 2002; Cuadrado *et al*, 2003). Some recent data indicate that, in leukaemic cells, APL action is mediated, at least in part, through Fas/CD95 cell death receptor, a member of the tumour necrosis factor (TNF) receptor family (Gajate *et al*, 2003). Depending on the cell system, APL induces either a very rapid apoptotic death without previous cell cycle arrest, or causes a block in G1 and/or a delay in the progression from S to G2/M phases of the cell cycle (Erba *et al*, 1999, 2002; Garcia-Fernandez *et al*, 2002). As part of its antitumoral action in leukaemic cells, APL inhibits the activity of ornithine decarboxylase (ODC), an enzyme responsible for polyamine biosynthesis and involved in cellular transformation (Paasinen-Sohns and Holtta, 1997; Erba *et al*, 1999; Iwata *et al*, 1999), and causes a reduction in vascular endothelial growth factor (VEGF) secretion and downregulation of its receptor, VEGFR-1 (flt-1), involved in the process of vascularisation and growth of certain tumours (Taraboletti *et al*, 2002; Broggini *et al*, 2003). In addition, it has been described that the APL closely related homologue didemnin B inhibits protein synthesis (Crampton *et al*, 1984) and may interact with elongation factor 1*α* (Crews *et al*, 1994) and palmitoyl thioesterase (Crews *et al*, 1996). However, no primary target for APL has been defined to date.

Drug resistance is a major obstacle to successful chemotherapy, and occurs to most commonly used antitumoral drugs as well as with the new target-oriented anticancer agents. As APL represents a novel antitumoral compound currently undergoing clinical studies, little is known about the possible mechanisms of intrinsic or acquired resistance to this drug. Considering the clinical interest of APL, we set out a project to obtain cell lines resistant to the compound. This paper describes the establishment and characterisation of a human carcinoma cell line, HeLa-APL, made resistant to APL after continuous exposure to increasing concentrations of the drug. Although HeLa-APL-resistant cells represent an *in vitro* model that is far from human tumour physiology, they can be potentially useful in the study of the mechanism of action of APL in tumour cells, in the selection of non-crossresistant APL structural analogues, and even to investigate and develop methods to prevent resistance to this drug *in vivo*.

## MATERIALS AND METHODS

### Reagents and antibodies

Aplidin™ (C_57_H_87_N_7_O_15_, MW: 1109.6), aplidin-dimethylaminocoumarin (APL-dmac) (C_62_H_89_N_7_O_15_, MW: 1172.41), didemnin B (C_57_H_89_N_7_O_15_, MW: 1112.354) and tamandarin A (C_54_H_85_N_7_O_14_, MW: 1056.291) were manufactured at PharmaMar, SA. Stock solutions (1 mg ml^−1^ in DMSO) were prepared and stored at −20°C. All other reagents used in this study were of molecular biology grade. Sulphorhodamine B (SRB), trizma, hoechst-33342, propidium iodide, paclitaxel, etoposide, camptothecin, adriamycin, vinblastine, cisplatin, 5-azacytidine, 6-thioguanine, fludarabine, 6-mercaptopurine, melphalan, chlorambucil, mitoxantrone and amsacrine were purchased from Sigma (St Louis, MO, USA). Calcein-AM was purchased from Calbiochem (Cambridge, MA, USA). DMEM, penicillin, streptomycin and foetal calf serum were purchased from Invitrogen (Carlsbad, CA, USA).

Anti-JNK1 (sc-474), anti-ERK2 (sc-154), anti-p38 MAPK (sc-535), anti-PARP (sc-7150), anti-(pro)caspase-3 (sc-7148) and anti-nPKC-*δ* (sc-937) polyclonal antibodies were purchased from Santa Cruz Biotechnology (Santa Cruz, CA, USA). Anti-phospho-p38 (#9211), anti-phospho-JNK (#9251), anti-phospho-PKC-*δ* (Thr505) (#9374), anti-phospho-PKC-*δ* (#9376) (Ser643) (polyclonal) and anti-phospho-ERK (#9106) (monoclonal) antibodies were purchased from Cell Signaling Technologies, Inc (Beverly, MA, USA). Anti-p170pgp/MDR (#MS-660-P0) monoclonal antibody was purchased from Neo Markers (Fremont, CA, USA).

### Cell culture

HeLa, HeLa-APL, LoVo and LoVo-dox (Grandi *et al*, 1986) cells were maintained in DMEM supplemented with 10% FCS and 100 U ml^−1^ penicillin and streptomycin at 37°C and 5% CO_2_.

### Microscopy

Cells were stained with APL-dmac, Calcein-AM or Hoechst-33342 by direct addition of the fluorescent compounds in the cell culture dishes, incubated at 37°C for the times indicated, washed in PBS and examined by fluorescence microscopy.

### Determination of cell survival

Cells were washed twice with PBS, fixed for 15 min in 1% glutaraldehyde solution, rinsed twice in PBS, and stained in 0.4% SRB solution for 30 min at room temperature. Cells were then rinsed several times with 1% acetic acid solution and air-dried. Sulphorhodamine B was then extracted in 10 mM trizma base solution and the absorbance measured at 490 nm. Cell survival was expressed as percentage of control cell growth.

### Flow cytometry analysis

The ploidy determination of nuclei was estimated by flow cytometry DNA analysis. Cells were washed in PBS, fixed by dropwise addition of 1 ml of cold 70% ethanol and allowed to stand on ice for 10 min. Cell pellet was resuspended in 200 *μ*l of PBS containing 10 *μ*g ml^−1^ RNAse A and incubated at 37°C for 30 min. Propidium iodide (200 *μ*g ml^−1^) was added and the DNA content per nucleus was evaluated in a FACscan flow cytometer (Becton-Dickinson). For the analysis, only signals from single nuclei were considered (10^4^ nuclei assay^−1^).

### Western blotting

Cells were washed in PBS, collected and resuspended in lysis buffer (20 mM Tris–HCl (pH 7.5), 150 mM NaCl, 1% (v v^−1^) Nonidet P-40, 2 mM EDTA, 1 mM PMSF, 10 *μ*g ml^−1^ aprotinin and 10 *μ*g ml^−1^ leupeptin) and kept on ice for 15 min. Cell extracts were cleared by microcentrifugation at 14 000 **g** for 30 min at 4°C. Equal amounts of extracts were resolved in SDS–PAGE and electroblotted to activated PVDF membranes (Immobilon-P, Millipore) following standard techniques (Sambrook *et al*, 1989). Membranes were sequentially probed with primary and appropriate secondary (horseradish-peroxidase-conjugated) antibodies following the manufacturer's instructions. Antibody–antigen complexes were detected using the ECL system (Amersham Biosciences, NJ, USA).

### Maldi TOF mass spectrometry

To detect minute amounts of APL present in the cellular extracts, Matrix-Assisted Laser Desorption/Ionisation-Time Of Flight Mass Spectrometry (Maldi-TOF MS) technique was used. Cell cultures were treated with vehicle or APL at the concentrations and times indicated in the text, washed with PBS and harvested by centrifugation at 6000 r.p.m. for 5 min at 4°C. Cell pellets were washed with PBS and extracted with cold methanol. Equal amounts of extract were applied on a template, air dried, mixed with 0.2–0.5 *μ*l matrix (10 mg ml^−1^ dihydroxy benzoic acid in water :acetonitrile mix, 1% trifluoroacetic acid) and air dried. Analyses were performed on a Voyager DE Pro (PerSeptive Biosystems, Framingham, MS, USA). Mass spectra were averaged over 100–200 laser shots from a nitrogen laser (337 nm) operating in a linear mode. Ionised molecules were accelerated with a voltage of 20 kV. The following peptides were used as external markers: intact human insulin, human insulin alpha-chain, human insulin beta-chain and cytochrome *c*.

## RESULTS

**Figure 2:**
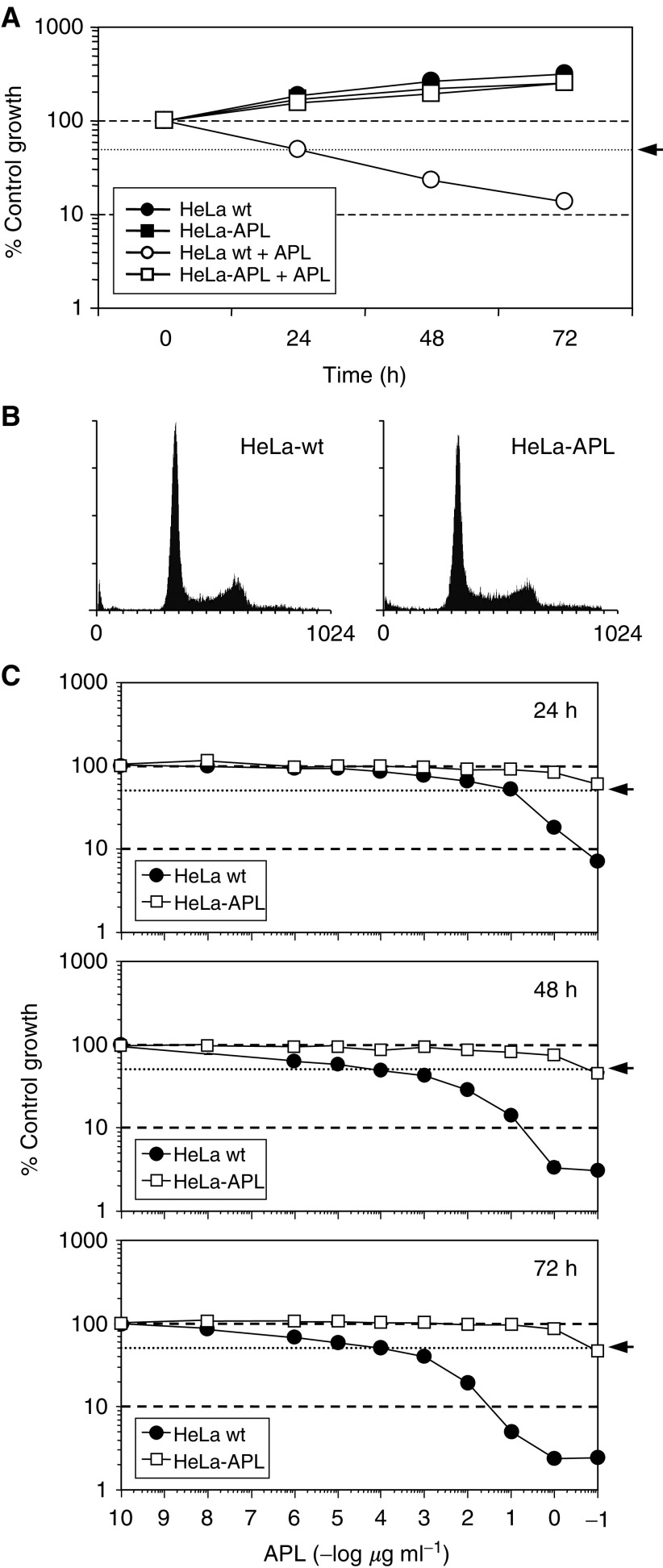
Comparison of Hela wt and Hela APL cells. (**A**) Time-course analysis of HeLa wt and HeLa-APL cell growth in the absence (vehicle) or presence of 450 nM APL. Cells were treated with APL for the indicated times and cell survival determined by sulphorhodamine B colorimetric assay. Results are expressed as percentage control growth. (**B**) Cell cycle distribution of exponentially growing HeLa wt and HeLa-APL cells, as analysed by flow cytometry. (**C**) Relative sensitivity of HeLa wt and HeLa-APL cells to APL. Cells were untreated (vehicle) or treated with the indicated concentrations of APL for 24, 48 and 72 h, and cell survival was determined by sulphorhodamine B colorimetric assay. Results are expressed as mean percentage of control growth ± s.e.m. (bars) of three independent experiments. Arrows indicate 50% control growth.

HeLa cells were selected because of its high sensitivity to APL among several cell lines tested in our laboratory. HeLa cells were continuously exposed to increasing concentrations of APL over a period of more than 1 year. Initial treatment started at 0.5 nM (aprox. five times the IC_50_ at 72 h) and was stepwise increased until 450 nM was reached. Viable cells surviving at this proapoptotic concentration were established and designated HeLa-APL. The morphology and growth features of HeLa-APL cell line were comparable to those of the parental wild-type cells, showing a comparable doubling time (about 24 h while exponentially growing). In the presence of 450 nM APL, HeLa-APL cells showed a rather delayed growth (Figure 2A

). By flow cytometric analysis after DNA staining with propidium iodide, log-phase HeLa-APL cells did not present apparent alterations in the cell cycle parameters as compared to the parental HeLa cells (Figure 2B). Using the SRB growth inhibition assay, HeLa-APL showed stable >1000-fold increase in the relative resistance factor to APL at the IC_50_ level (Figure 2C). Resistance was not reversed while maintaining the cell line in drug-free medium for at least 3 months (not shown).

**Figure 3:**
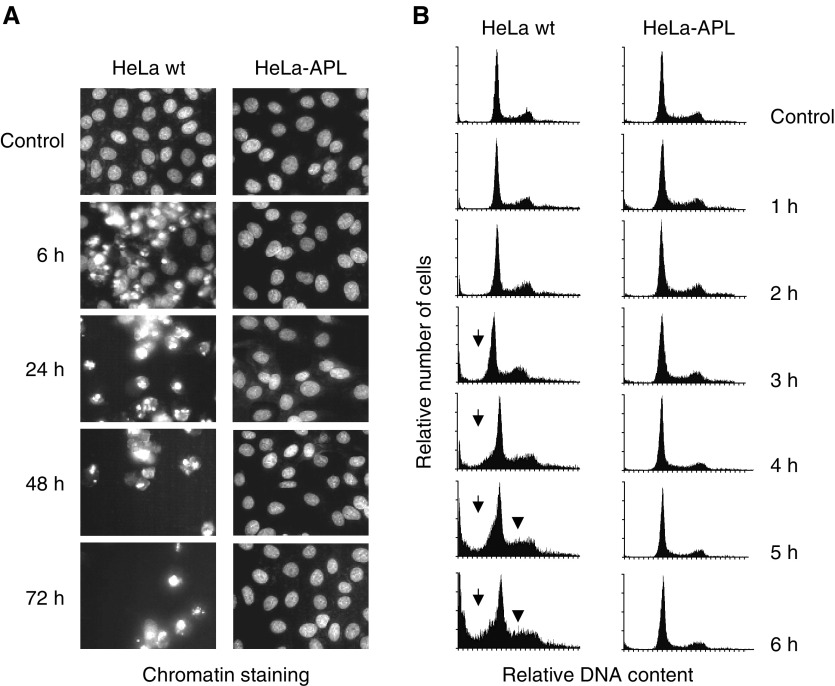
Time-dependent effects of APL on chromatin integrity and DNA ploidy. HeLa wt and HeLa-APL cells were treated with vehicle alone or 450 nM APL for the indicated time periods. (**A**) Phenotypic changes induced by APL as analysed by fluorescence microscopy after chromatin staining with Hoechst 33342. (**B**) Time course of APL-induced apoptosis as analysed by flow cytometry. Sub-Gl, hypodiploid population (arrows) and delayed progression through S phase (arrowheads) are indicated.

Treatment of parental HeLa cells with 450 nM APL induced progressive chromatin condensation and the appearance of typical apoptotic bodies (as revealed by fluorescent staining of chromatin with permeable Hoechst-33342) that resulted in massive cell death as early as 6 h post-treatment (Figure 3A

, left). By flow cytometry analysis, it was correlated with a rapid and progressive accumulation of hypodiploid cells in the sub-G1 region (apoptotic cells, arrows) and a delayed progression through S to G2/M phase of the cell cycle (arrow heads) (Figure 3B, left). When HeLa-APL cells were treated with APL under the same conditions, neither signs of apoptotic population (Figure 3A, right) nor perturbations in the cell cycle (Figure 3B, right) were observed at any of the time points studied.

**Figure 4:**
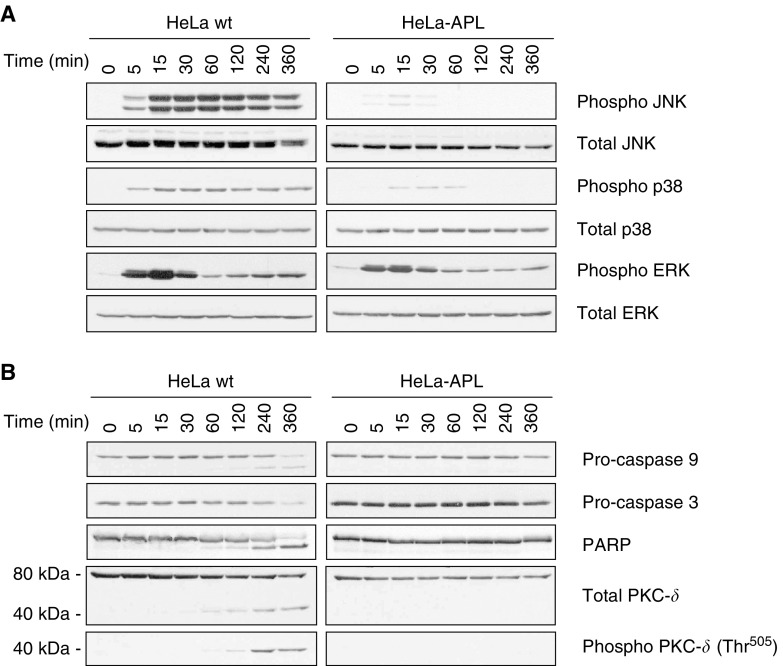
Time-dependent effects of APL on MAPKs and caspase cascade. HeLa wt and HeLa-APL cells were treated with vehicle alone or 450 nM APL for the indicated time periods (min) and JNK, p38 MAPK and ERK (p42/p44) (**A**) or caspase-9, caspase-3, PARP and PKC-*δ* (**B**) protein status analysed by Western blotting. Membranes were reprobed with antibodies against total JNK, p38 MAPK, ERK (p42/p44) and PKC-*δ* for normalisation.

Aplidin™ has been shown to rapidly induce the mitochondrial apoptotic pathway via rapid and persistent activation of JNK and p38 MAPK (Garcia-Fernandez *et al*, 2002; Cuadrado *et al*, 2003). Since APL appeared to be unable to induce apoptosis in HeLa-APL-resistant cells, we studied the molecular effects of APL in both cell systems. Wild-type and resistant cells were treated with 450 nM APL for different times and the expression of proteins involved in the induction and execution of apoptosis were analysed by Western blotting. Results showed that, in parental HeLa cells, APL induced rapid (around 5 min) and persistent JNK and p38 MAPK phosphorylation, as expected. By contrast, in HeLa-APL-resistant cells, only a very weak and transient JNK and p38 MAPK activation was observed after drug treatment, indicating that sustained activation of stress-activated MAPKs plays an essential role in mediating the cytotoxic effects of APL in tumour cells (Figure 4A

). It was also previously reported that APL activates ERK (p42/p44-MAPK) phosphorylation, in a time-dependent biphasic manner in HeLa parental cells. Interestingly, first peaking at 15 min post-treatment appeared similarly in both cell systems, while second peaking at around 6 h post-treatment was absent in HeLa-APL-resistant cells (Figure 4A). Remarkably, no changes in the level of protein expresion were detected after re-probing blots against total JNK and p38 MAPK in either case, indicating that APL induced phosphorylation of pre-existing MAPKs and not promoting *de novo* protein synthesis. Immunoblotting analysis of proteins involved in the initiation and execution of apoptosis showed activation of caspase-9 and -3, inactivation of PARP (emergence of cleaved 85 kDa fragment) and concomitant cleavage and phosphorylation of PKC-*δ* (40 kDa proteolytic product phosphorylated at Thr^505^). Consistently with previously shown morphological appearance and cell cycle analysis results, none of these molecular events were observed in HeLa-APL-resistant cells, indicating the complete absence of apoptotic response after treatment with supraphysiologic concentrations of APL (Figure 4B).

**Figure 5:**
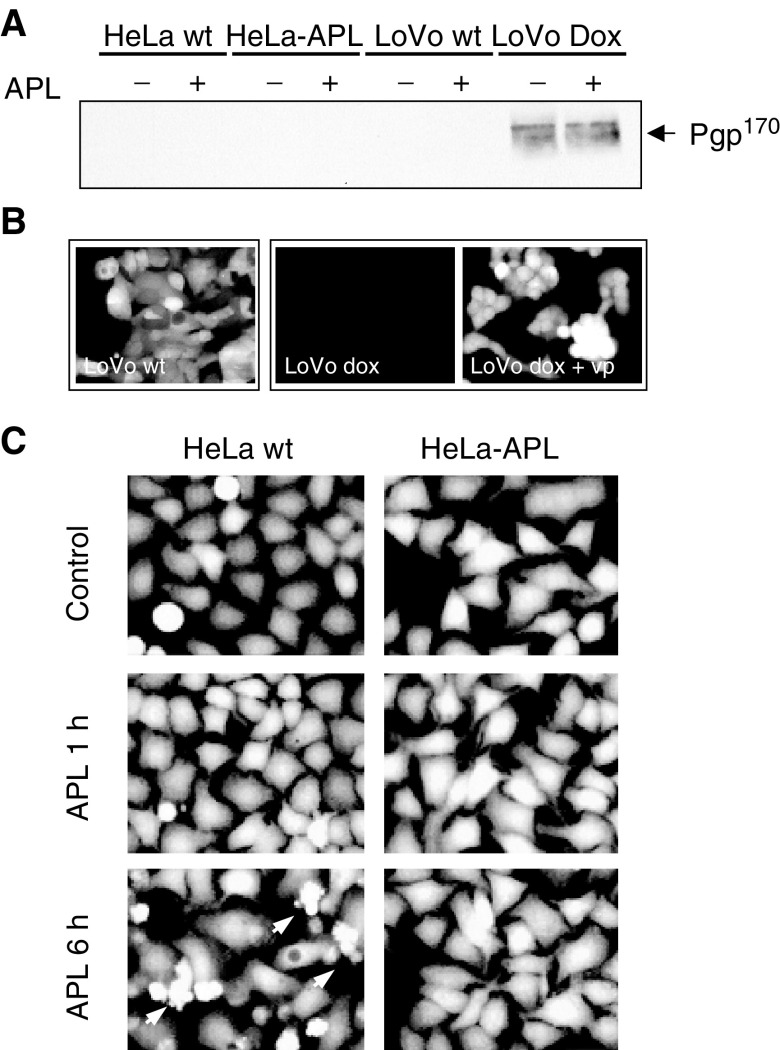
Role of P-glycoprotein (Pgp^170^) in HeLa-APL acquired resistance. (**A**) HeLa wt, HeLa-APL, LoVo and LoVo-dox cells were treated with vehicle alone or 450 nM APL for 6 h and Pgp^170^ protein expression analysed by Western blotting. (**B**) LoVo wt, LoVo-dox and LoVo-dox cells pretreated with 50 *μ*M verapamil (LoVo-dox + vp), were loaded with Calcein-AM for 1 h and then analysed by fluorescence microscopy. (**C**) HeLa wt and HeLa-APL cells were treated with vehicle alone or 450 nM APL for the indicated time periods, loaded with Calcein-AM for 1 h and analysed by fluorescence microscopy.

**Figure 6:**
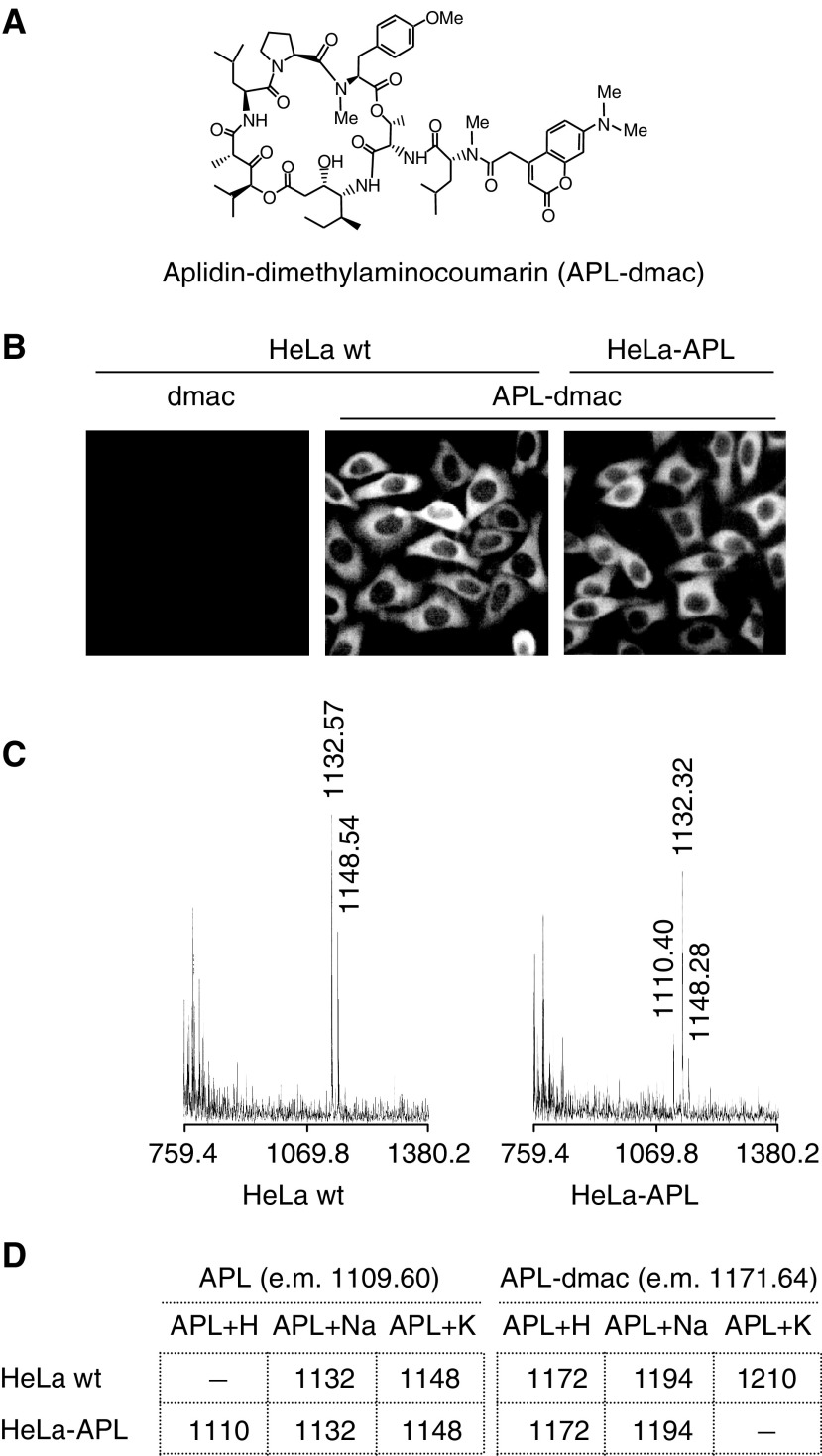
Intracellular accumulation of APL in HeLa wt and HeLa-APL cells. (**A**) Molecular structure of fluorescent derivative APL-dimethylaminocoumarin (APL-dmac). (**B**) Hela wt and HeLa-APL cells were treated with vehicle alone or 450 nM APL-dmac for 1 h and then analysed by fluorescence microscopy. Dimethylaminocoumarin was used as background control in HeLa wt cells (dmac). Representative images are shown. (**C**) Maldi-TOF analysis of APL in HeLa wt and HeLa-APL cells after treatment with vehicle or 450 nM APL for 1 h. For clarity, only chromatograms corresponding to APL are shown. Exact masses of peaks corresponding to APL+H, APL+Na and APL+K ionised forms are indicated (when detected). (**D**) Summary of APL and APL-dmac ionised forms detected in HeLa wt and HeLa-APL cells, e.m., exact mass.

Decreased drug accumulation is often associated with resistance of tumour cells to anticancer drugs and frequently involves overexpression of drug efflux proteins, P-glycoprotein (Pgp^170^) being the most common pump related to drug resistance (Germann, 1996; Gottesman *et al*, 2002). The expression of Pgp^170^/MDR was studied in both parental and resistant cells by Western blotting. As a positive control, we used the multidrug-resistant cell line LoVo-Dox, a colorectal carcinoma cell line overexpressing Pgp^170^ after long-term exposure to doxorubicin. LoVo parental cells, which do not express Pgp^170^, were also included (LoVo wt). As shown in Figure 5A

, no detectable expression of Pgp^170^ was observed in HeLa or HeLa-APL cells under either untreated or APL-treated conditions (6 h). By contrast, Pgp^170^ protein was strongly expressed in LoVo-Dox cells. Its expression was not altered by APL treatment. The absence of a Pgp^170^ acting in HeLa-APL cells was further confirmed by the use of the permeable substrate calcein-AM, which, after intracellular cleavage by unspecific esterases, accumulates in the cell and become impermeable and highly fluorescent. As a control system, we used again LoVo and LoVo-Dox cells. Calcein-AM accumulates in LoVo-sensitive cells, but was excluded from resistant LoVo-Dox cells. Pre-incubation of cell cultures with 50 *μ*M verapamil (vp), a potent Pgp^170^ inhibitor, resulted in accumulation of calcein-AM in LoVo-Dox-resistant cells to an extent similar to that observed in sensitive LoVo cells, demonstrating the effectiveness of the fluorescent marker to assess for Pgp^170^ action (Figure 5B). Accumulation of fluorescent calcein-AM was consistent in both HeLa parental and HeLa-APL-resistant cells (Figure 5C). Furthermore, APL treatment did not modify the fluorescent pattern in either case, even after the apoptotic programme became apparent in HeLa-sensitive cells (Figure 5C, arrows). However, since other efflux pumps may contribute to decreased accumulation of APL in HeLa-APL cells (Borst *et al*, 2000; Gottesman *et al*, 2002), we used a fluorescent APL derivative (APL-dmac, see Materials and methods) (Figure 6A

) with cytotoxic activity similar to that of APL (not shown) (Ding *et al*, 2001). Results showed that internalisation and accumulation of APL-dmac in the cytoplasmic compartment was consistent in both cell systems (Figure 6B). Background fluorescence accumulation was assessed by using dimethylaminocoumarin alone (Figure 6B, dmac), which showed no detectable fluorescent signal under the same conditions. Aplidin™-dmac remained internalised after long-term treatment (not shown). To more precisely demonstrate the integrity of the drug after intracellular accumulation, we used highly sensitive Maldi-TOF mass spectrometry (Marvin *et al*, 2003). The presence of APL in both parental and HeLa-APL-resistant cell systems after 1 h exposure was confirmed, as demonstrated by the detection of the different ionised forms of APL, that is, ∼1110 for APL+H, ∼1132 for APL+Na and ∼1148 for APL+K. (Figure 6C and D). Consistently, APL-dmac was also detected in both cell lines (Figure 6D).

**Figure 7:**
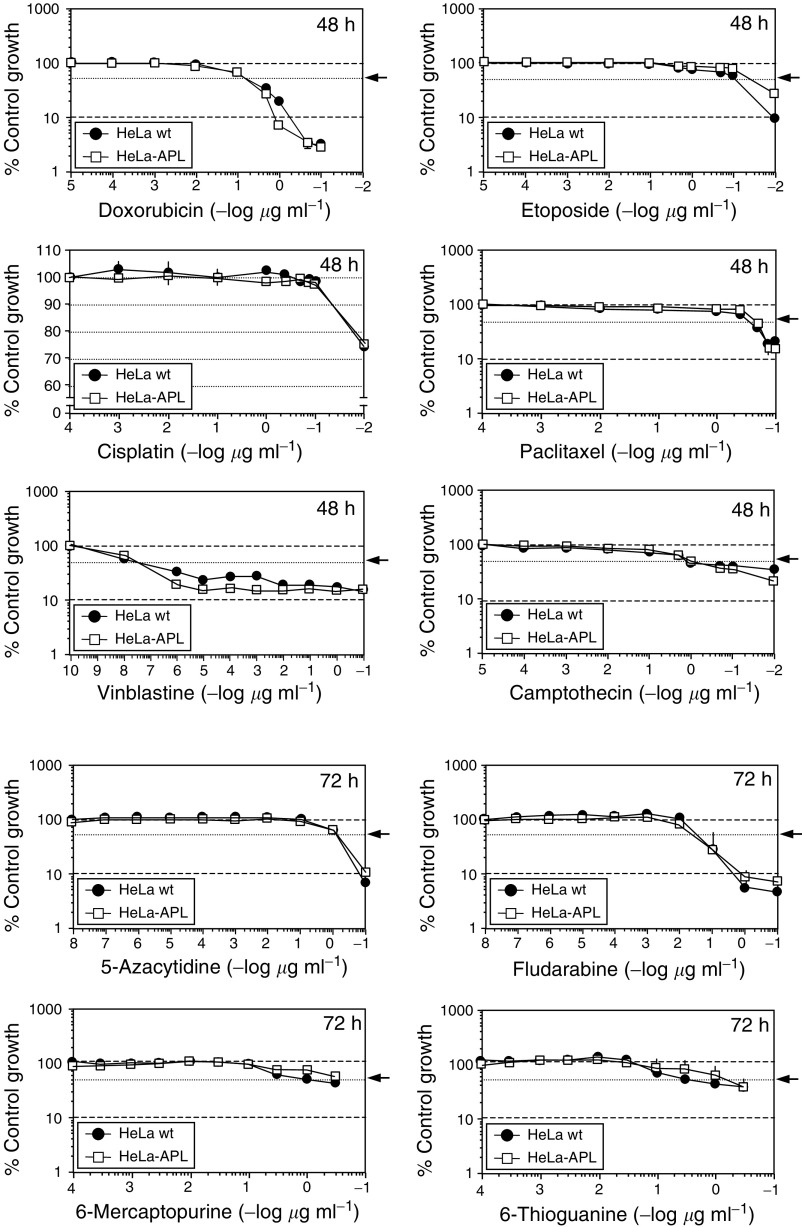
Relative sensitivity of HeLa wt and HeLa-APL cells to a panel of anticancer drugs. Triplicate plates of HeLa wt and HeLa-APL cells were exposed to vehicle alone or the indicated concentrations of doxorubicin, etoposide, cisplatin, paclitaxel, vinblastine, camptothecin, 5-azacytidine, fludarabine, 6-mercaptopurine and 6-thioguanine for 48 or 72 h (as indicated). Cell survival was determined by sulphorhodamine B colorimetric assay. Results are expressed as percentage control growth ± s.e.m. Arrows indicate 50% control growth.

**Figure 8:**
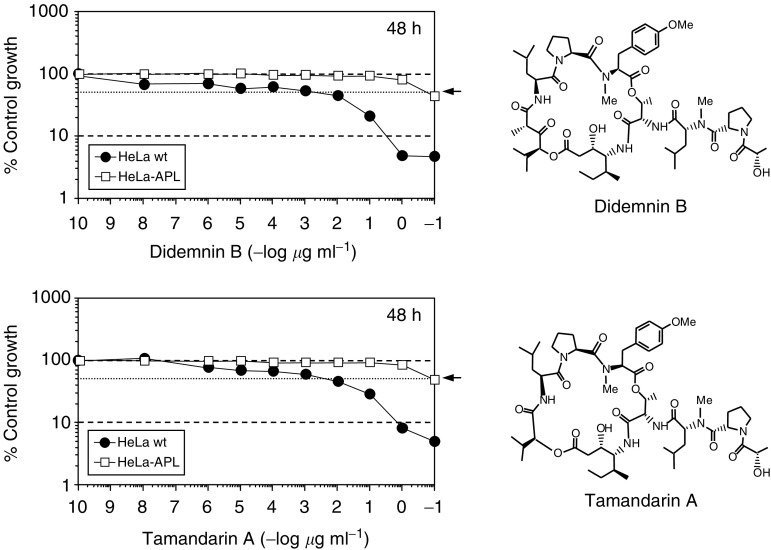
Relative sensitivity of HeLa wt and HeLa-APL cells to APL structural analogues. Triplicate plates of HeLa wt and HeLa-APL cells were exposed to vehicle alone or the indicated concentrations of Didemnin B and Tamandarin A for 48 h and cell survival was determined by sulphorhodamine B colorimetric assay. Results are expressed as percentage control survival ± s.e.m. Molecular structures of Didemnin B and Tamandarin A are shown. Arrows indicate 50% control growth.

The pattern of sensitivity of parental and resistant cells and crossresistance were determined for a series of standard anticancer drugs (cisplatin, etoposide, doxorubicin, paclitaxel, vinblastine, camptothecin, 5-azacytidine, 6-thioguanine, fludarabine and 6-mercaptopurine) and two additional members of the didemnin family with cytotoxic activity comparable to that of APL (Vervoort and Fenical, 2000; Joullie *et al*, 2003), didemnin B and tamandarin A. Notably, no significant crossresistance was observed with any of the standard anticancer drugs tested, as determined by IC_50_ values in the SRB cell growth assay (Figure 7

). On the other hand, comparable crossresistance was observed with the APL structural analogues didemnin B and tamandarin A (Figure 8

).

## DISCUSSION

A human carcinoma cell line with acquired resistance to APL, a new anticancer drug currently undergoing extensive phase II clinical studies, has been established by continuous exposure to stepwise increasing concentrations of the drug. HeLa-APL cells showed more than 1000-fold stable resistance to APL than the parental HeLa cells at the IC_50_ value. Clinical resistance may be demonstrably more subtle, reflecting only 2–5-fold changes, but differences in *in vitro* models are more dramatic due to the intensive artificial selection for resistant subclones. When comparing the chemosensitivity of HeLa-APL cells to other commonly used anticancer drugs, no significant crossresistance was observed with doxorubicin, cisplatin, etoposide, paclitaxel, vinblastine, camptothecin, 5-azacytidine, 6-thioguanine, fludarabine and 6-mercaptopurine (as well as amsacrine, melphalan, chlorambucil or mitoxantrone, not shown). A trend for crossresistance for APL and 2′,2′-difluorodeoxycytidine (gemcitabine) in human leukaemia samples has been recently reported (Bresters *et al*, 2003), but not with other antimetabolites, as occurred herein with those tested in HeLa-APL cells (5-azacytidine, 6-thioguanine, fludarabine and 6-mercaptopurine). By contrast, HeLa-APL cells were equally resistant to didemnin B and tamandarin A, two closely related APL structural analogues, demonstrating that HeLa-APL-resistant cells have developed a specific resistance mechanism against didemnins (Vervoort and Fenical, 2000; Joullie *et al*, 2003). On the other hand, the lack of crossresistance with other anticancer agents may reflect differences in its mode of action or cellular pharmacology, and suggests the possibility to treat relapsed or resistant cancers to conventional therapies with APL and *vice versa*.

Drug resistance is often associated with decreased drug internalisation or increased drug efflux and can involve overexpression of drug efflux pumps (Germann, 1996; Gottesman *et al*, 2002). However, no Pgp^170^ protein expression or activity was observed in either parental or resistant cell line even after APL treatment. The participation of other mechanisms related to decreased drug accumulation was precluded given the following observations: (i) fluorescent APL accumulates to the same extent in both cell lines; (ii) conserved sensitivity to different commonly used cytotoxic drugs, most of them exported by Pgp^170^, MRP or other unspecific pumps (Gottesman *et al* (2002) and references therein); (iii) APL was detected by high-sensitivity mass spectrometry in both cell systems, an observation that, in addition, also rules out the possibility of a resistance mechanism due to changes in the intracellular metabolism of the drug.

We have previously shown that APL induces rapid and sustained activation of JNK and p38 MAPK in tumour cells, and this activation is pivotal to establish and maintain the apoptotic programme (Garcia-Fernandez *et al*, 2002; Cuadrado *et al*, 2003). The timing of JNK and p38 MAPK activation may be of crucial importance, being a persistent rather than transient activation associated with apoptosis (Chen *et al*, 1996a, 1996b; Brenner *et al*, 1997). Long-lasting stimulation of stress MAPKs has been described for different forms of stress-induced apoptosis (Xia *et al*, 1995; Chen *et al*, 1996b, 1999; Herr *et al*, 1997; Luo *et al*, 1998; Bacus *et al*, 2001). When comparing both cell lines, we found that, contrary to wild-type cells, in resistant HeLa-APL cells, APL treatment only induce a weak and transient activation of both JNK and p38 MAPK and fails to activate the downstream apoptotic cascade. This indicates the essential role of these two MAPKs in priming the apoptotic programme induced by the drug. It has been recently demonstrated that cells lacking JNK expression (jnk1/2^−/−^) are much less sensitive to APL than their normal counterparts, pointing out the crucial role of JNK in APL-induced apoptosis (Cuadrado *et al*, 2004). Previous observations suggested that the rapid activation of JNK and p38 MAPK is most probably due to an early cellular oxidative imbalance generated by the drug, as demonstrated by the protective effects of some antioxidants (Garcia-Fernandez *et al*, 2002; Cuadrado *et al*, 2003). The lack of MAPK activation upon drug treatment may indicate that resistant cells have developed a specific mechanism to overcome APL-mediated oxidative imbalance and the subsequent sustained activation of the stress cascade. Although no direct correlation between GSH content and APL sensitivity has been published to date, GSH would appear as a putative sensor for APL action that has to be further examined in HeLa-APL-resistant cells. Similar mechanisms have been reported previously (Whelan *et al*, 1989; Miyanishi *et al*, 2001; Cao *et al*, 2003). Concerning APL-mediated ERK activation, we previously concluded that, in general, it was not necessary for the cytotoxic action of the drug and that, most probably, it was serving a protective role against the insult caused by the drug (Garcia-Fernandez *et al*, 2002). In the present work, we demonstrate a similar early ERK activation in both parental and resistant HeLa-APL cells after drug treatment, confirming the protective role of this rapid and transient ERK activation. We were curious about the delayed ERK activation observed, and hypothetised that it could be due to late activation of the MEK-ERK pathway by PKC-*δ*, a link that has been previously reported (Ueda *et al*, 1996). Herein we show that in HeLa-APL-resistant cell line, APL, does not induce either PKC-*δ* or delayed ERK activation, further supporting the above-mentioned hypothesis.

Mitochondria and caspases have key roles in apoptosis and it was considered whether HeLa-APL cell line had subresponsive apoptotic pathways. We show herein that HeLa-APL cells had intact response to a number of caspase-dependent death inducers like doxorubicin, camptothecin, paclitaxel or etoposide, and therefore most likely had an intact apoptosis execution machinery, further indicating that the alteration was upstream of caspase activation.

In conclusion, the work presented herein describes the first example of the generation of a cell line that is resistant to APL, which is currently undergoing clinical trials. A number of potential mechanisms for the resistance was ruled out, including those involving altered drug transport, accumulation, metabolism or subresponsive apoptotic pathways. HeLa-APL cells represent a potentially useful tool in gaining insights into the mode of action of APL in tumour cells, in selecting non-crossresistant APL structural analogues, as well as to investigate and develop methods to prevent resistance to this drug.
